# Supplementary material for: Efficacy and Safety of Sugammadex for the Reversal of Rocuronium-Induced Neuromuscular Blockade in Patients with End-Stage Renal Disease: A Systematic Review and Meta-Analysis
Source: Medicina (Kaunas). 2021 Nov 17;57(11):1259. doi: 10.3390/medicina57111259 (PMC8622972; doi:10.3390/medicina57111259)
Supplement: Supplementary file 1 [file medicina-57-01259-s001.zip › medicina-1450388-supplementary.pdf]

Table S1: Search strategies for each database

No.	PubMed	Embase	Cochrane Library	Web of Science (Including ESCI)	Scopus	KoreaMed
#1	Renal Insufficiency[mesh:noexp] OR Acute Kidney Injury[mesh:noexp] OR Acute Kidney Tubular Necrosis[mesh:noexp] OR Chronic Renal Insufficiency[mesh:noexp] OR Chronic Kidney Failure[mesh:noexp]	('kidney failure'/de OR 'acute kidney failure'/exp OR 'acute kidney tubule necrosis'/exp OR 'kidney tubule necrosis'/exp OR 'chronic kidney failure'/exp OR 'end stage renal disease'/exp)	[mh ^"Renal Insufficiency"] OR [mh ^"Acute Kidney Injury"] OR [mh ^"Acute Kidney Tubular Necrosis"] OR [mh ^"Chronic Renal Insufficiency"] OR [mh ^"Chronic Kidney Failure"]	TS=("renal insufficienc*" OR "kidney insufficienc*" OR "kidney failur*" OR "renal failur*")	TITLE-ABS-KEY("renal insufficienc*" OR "kidney insufficienc*" OR "kidney failur*" OR "renal failur*")	renal OR kidney
#2	Renal insufficienc*[tiab] OR Kidney Insufficienc*[tiab] OR Kidney Failur*[tiab] OR Renal Failur*[tiab]	('renal insufficienc*':ti,ab,kw OR 'kidney insufficienc*':ti,ab,kw OR 'kidney failur*':ti,ab,kw OR 'renal failur*':ti,ab,kw)	("renal insufficienc*":ti,ab,kw OR "kidney insufficienc*":ti,ab,kw OR "kidney failur*":ti,ab,kw OR "renal failur*":ti,ab,kw)	TS=("acute kidney injur*" OR "acute renal injur*" OR "kidney acute failure*")	TITLE-ABS-KEY("acute kidney injur*" OR "acute renal injur*" OR "kidney acute failure*")	insufficiency OR insufficiencies OR failure OR failures OR injury OR injuries OR disease OR diseases OR disorder OR disorders OR necrosis OR necroses OR impairment OR impairments
#3	Acute Kidney Injur*[tiab] OR Acute Renal Injur*[tiab] OR "Kidney acute failure"[tiab] OR "Kidney acute failures"[tiab]	('acute kidney injur*':ti,ab,kw OR 'acute renal injur*':ti,ab,kw OR 'kidney acute failure*':ti,ab,kw)	("acute kidney injur*":ti,ab,kw OR "acute renal injur*":ti,ab,kw OR "kidney acute failure*":ti,ab,kw)	TS=("lower nephron nephros*" OR "acute renal tubular failure*" OR "kidney tubular epithelium necrosis" OR "necrotic tubulonephros*" OR "renal tubular cell necrosis" OR "tubular necrosis" OR "tubule necrosis" OR "tubulus necrosis")	TITLE-ABS-KEY("lower nephron nephros*" OR "acute renal tubular failure*" OR "kidney tubular epithelium necrosis" OR "necrotic tubulonephros*" OR "renal tubular cell necrosis" OR "tubular necrosis" OR "tubule necrosis" OR "tubulus necrosis")	sugammadex OR rocuronium OR neuromuscular OR cyclodextrins OR bridion OR esmeron OR zenuron
#4	"Lower Nephron Nephrosis"[tiab] OR "Lower nephron nephroses"[tiab] OR "acute renal tubular failure"[tiab] OR "acute renal tubular failures"[tiab] OR "kidney tubular epithelium necrosis"[tiab] OR	('lower nephron nephros*':ti,ab,kw OR 'acute renal tubular failure*':ti,ab,kw OR 'kidney tubular epithelium necrosis':ti,ab,kw OR 'necrotic tubulonephros*':ti,ab,kw OR 'renal tubular cell necrosis':ti,ab,kw	("lower nephron nephros*":ti,ab,kw OR "acute renal tubular failure*":ti,ab,kw OR "kidney tubular epithelium necrosis":ti,ab,kw OR "necrotic tubulonephros*":ti,ab,kw OR "renal tubular	TS=("chronic kidney disease*" OR "chronic renal disease*" OR "chronic kidney disorder*" OR "chronic nephropath*" OR "kidney chronic failure*" OR "chronic disease kidney function" OR "chronic	TITLE-ABS-KEY("chronic kidney disease*" OR "chronic renal disease*" OR "chronic kidney disorder*" OR "chronic nephropath*" OR "kidney chronic failure*" OR "chronic disease kidney	#1 AND #2 AND #3

	"necrotic tubulonephrosis"[tiab] OR "renal tubular cell necrosis"[tiab] OR tubular necrosis[tiab] OR tubule necrosis[tiab] OR tubulus necrosis[tiab]	OR 'tubular necrosis':ti,ab,kw OR 'tubule necrosis':ti,ab,kw OR 'tubulus necrosis':ti,ab,kw)	cell necrosis":ti,ab,kw OR "tubular necrosis":ti,ab,kw OR "tubule necrosis":ti,ab,kw OR "tubulus necrosis":ti,ab,kw)	diseases kidney function")	function" OR "chronic diseases kidney function")
#5	Chronic Kidney Disease*[tiab] OR Chronic Renal Disease*[tiab] OR chronic kidney disorder*[tiab] OR chronic nephropath*[tiab] OR "kidney chronic failure"[tiab] OR "Kidney chronic failures"[tiab] OR "chronic disease kidney function"[tiab] OR "chronic diseases kidney function"[tiab]	('chronic kidney disease*':ti,ab,kw OR 'chronic renal disease*':ti,ab,kw OR 'chronic kidney disorder*':ti,ab,kw OR 'chronic nephropath*':ti,ab,kw OR 'kidney chronic failure*':ti,ab,kw OR 'chronic disease kidney function':ti,ab,kw OR 'chronic diseases kidney function':ti,ab,kw)	("chronic kidney disease*":ti,ab,kw OR "chronic renal disease*":ti,ab,kw OR "chronic kidney disorder*":ti,ab,kw OR "chronic nephropath*":ti,ab,kw OR "kidney chronic failure*":ti,ab,kw OR "chronic disease kidney function":ti,ab,kw OR "chronic diseases kidney function":ti,ab,kw)	TS=("end stage kidney disease*" OR "end stage renal disease*" OR "end stage kidney failure*" OR "end stage renal dysfunction*" OR "end stage renal failure*" OR "end stage renal impairment*" OR "end stage renal insufficienc*" OR "end-stage kidney disease" OR "end-stage kidney failure" OR "end-stage renal disease" OR "end-stage renal failure" OR esrd OR "stage 5 kidney disease" OR "stage 5 renal disease")	TITLE-ABS-KEY("end stage kidney disease*" OR "end stage renal disease*" OR "end stage kidney failure*" OR "end stage renal dysfunction*" OR "end stage renal failure*" OR "end stage renal impairment*" OR "end stage renal insufficienc*" OR "end-stage kidney disease" OR "end-stage kidney failure" OR "end-stage renal disease" OR "end-stage renal failure" OR esrd OR "stage 5 kidney disease" OR "stage 5 renal disease")
#6	end stage kidney disease*[tiab] OR end stage renal disease*[tiab] OR end stage kidney failure*[tiab] OR end stage renal dysfunction*[tiab] OR end stage renal failure*[tiab] OR end stage renal impairment*[tiab] OR end stage renal insufficienc*[tiab] OR "end-stage kidney disease"[tiab] OR "end-stage kidney failure"[tiab] OR "end-stage renal disease"[tiab] OR "end-stage renal failure"[tiab] OR ESRD[tiab] OR "stage 5 kidney disease"[tiab]	('end stage kidney disease*':ti,ab,kw OR 'end stage renal disease*':ti,ab,kw OR 'end stage kidney failure*':ti,ab,kw OR 'end stage renal dysfunction*':ti,ab,kw OR 'end stage renal failure*':ti,ab,kw OR 'end stage renal impairment*':ti,ab,kw OR 'end stage renal insufficienc*':ti,ab,kw OR 'end-stage kidney disease':ti,ab,kw OR 'end-stage kidney failure':ti,ab,kw OR 'end-stage renal disease':ti,ab,kw OR 'end-stage renal failure':ti,ab,kw OR esrd:ti,ab,kw OR 'stage 5 kidney	("end stage kidney disease*":ti,ab,kw OR "end stage renal disease*":ti,ab,kw OR "end stage kidney failure*":ti,ab,kw OR "end stage renal dysfunction*":ti,ab,kw OR "end stage renal failure*":ti,ab,kw OR "end stage renal impairment*":ti,ab,kw OR "end stage renal insufficienc*":ti,ab,kw OR "end-stage kidney disease":ti,ab,kw OR "end-stage kidney failure":ti,ab,kw OR "end-stage renal disease":ti,ab,kw OR "end-stage renal failure":ti,ab,kw OR	#1 OR #2 OR #3 OR #4 OR #5	#1 OR #2 OR #3 OR #4 OR #5

	OR "stage 5 renal disease"[tiab]	disease':ti,ab,kw OR 'stage 5 renal disease':ti,ab,kw)	esrd:ti,ab,kw OR "stage 5 kidney disease":ti,ab,kw OR "stage 5 renal disease":ti,ab,kw)		
#7	#1 OR #2 OR #3 OR #4 OR #5 OR #6	#1 OR #2 OR #3 OR #4 OR #5 OR #6	#1 OR #2 OR #3 OR #4 OR #5 OR #6	TS=("neuromuscular block*" OR "myoneural blockade" OR "nerve muscle block" OR "neuromuscular inhibition" OR "neuromuscular receptor blocking" OR "neuromuscular transmission blocking")	TITLE-ABS-KEY("neuromuscular block*" OR "myoneural blockade" OR "nerve muscle block" OR "neuromuscular inhibition" OR "neuromuscular receptor blocking" OR "neuromuscular transmission blocking")
#8	Neuromuscular Blockade[mesh] OR Sugammadex OR gamma-Cyclodextrins[mesh] OR rocuronium	('neuromuscular blocking'/exp OR 'sugammadex'/exp OR 'gamma cyclodextrin derivative'/exp OR 'rocuronium'/exp)	[mh "Neuromuscular Blockade"] OR Sugammadex OR [mh "gamma-Cyclodextrins"] OR rocuronium	TS=("gamma-cyclodextrins" OR "gamma cyclodextrins")	TITLE-ABS-KEY("gamma-cyclodextrins" OR "gamma cyclodextrins")
#9	Neuromuscular blockade[tiab] OR Neuromuscular Block[tiab] OR neuromuscular blocking[tiab] OR myoneural blockade[tiab] OR nerve muscle block[tiab] OR neuromuscular inhibition[tiab] OR "neuromuscular receptor blocking"[tiab] OR "neuromuscular transmission blocking"[tiab]	('neuromuscular block*':ti,ab,kw OR 'myoneural blockade':ti,ab,kw OR 'nerve muscle block':ti,ab,kw OR 'neuromuscular inhibition':ti,ab,kw OR 'neuromuscular receptor blocking':ti,ab,kw OR 'neuromuscular transmission blocking':ti,ab,kw)	("neuromuscular block*":ti,ab,kw OR "myoneural blockade":ti,ab,kw OR "nerve muscle block":ti,ab,kw OR "neuromuscular inhibition":ti,ab,kw OR "neuromuscular receptor blocking":ti,ab,kw OR "neuromuscular transmission blocking":ti,ab,kw)	TS=("6-perdeoxy-6-per(2-carboxyethyl)thio-gamma-cyclodextrin sodium salt" OR bridion OR "org 25969" OR org25969)	TITLE-ABS-KEY("6-perdeoxy-6-per(2-carboxyethyl)thio-gamma-cyclodextrin sodium salt" OR bridion OR "org 25969" OR org25969)
#10	"gamma-Cyclodextrins" OR "gamma Cyclodextrins"	('gamma-cyclodextrins' OR 'gamma cyclodextrins')	("gamma-cyclodextrins" OR "gamma cyclodextrins")	TS=("1-(17-(acetoyl)-3-hydroxy-2-(4-morpholinyl)androstan-16-yl)-1-(2-propenyl)pyrrolidinium" OR rocuronium OR "pyrrolidinium, 1-((2beta,3alpha,5alpha,16beta,17beta)-17-(acetyloxy)-3-hydroxy-2-(4-morpholinyl)androstan-16-yl)-1-(2-propenyl)-, bromide"	TITLE-ABS-KEY("1-(17-(acetoyl)-3-hydroxy-2-(4-morpholinyl)androstan-16-yl)-1-(2-propenyl)pyrrolidinium" OR rocuronium OR "pyrrolidinium, 1-((2beta,3alpha,5alpha,16beta,17beta)-17-(acetyloxy)-3-hydroxy-2-(4-morpholinyl)androstan-16-yl)-1-(2-

				OR esmeron OR esmerone OR "org 9426" OR org9426 OR zemuron)	propenyl)-, bromide" OR esmeron OR esmerone OR "org 9426" OR org9426 OR zemuron)
#11	"6-perdeoxy-6-per(2-carboxyethyl)thio-gamma-cyclodextrin sodium salt" OR Bridion OR "Org 25969" OR Org25969	('6-perdeoxy-6-per(2-carboxyethyl)thio-gamma-cyclodextrin sodium salt' OR bridion OR 'org 25969' OR org25969)	("6-perdeoxy-6-per(2-carboxyethyl)thio-gamma-cyclodextrin sodium salt" OR bridion OR "org 25969" OR org25969)	#7 OR #8 OR #9 OR #10	#7 OR #8 OR #9 OR #10
#12	"1-(17-(acetoyl)-3-hydroxy-2-(4-morpholinyl)androstan-16-yl)-1-(2-propenyl)pyrrolidinium" OR rocuronium OR "pyrrolidinium, 1-((2beta,3alpha,5alpha,16beta,17beta)-17-(acetyloxy)-3-hydroxy-2-(4-morpholinyl)androstan-16-yl)-1-(2-propenyl)-, bromide" OR Esmeron OR Esmerone OR ORG-9426 OR "ORG 9426" OR Org9426 OR Zemuron	('1-(17-(acetoyl)-3-hydroxy-2-(4-morpholinyl)androstan-16-yl)-1-(2-propenyl)pyrrolidinium' OR rocuronium OR 'pyrrolidinium, 1-((2beta,3alpha,5alpha,16beta,17beta)-17-(acetyloxy)-3-hydroxy-2-(4-morpholinyl)androstan-16-yl)-1-(2-propenyl)-, bromide' OR esmeron OR esmerone OR 'org 9426' OR org9426 OR zemuron)	("1-(17-(acetoyl)-3-hydroxy-2-(4-morpholinyl)androstan-16-yl)-1-(2-propenyl)pyrrolidinium" OR rocuronium OR "pyrrolidinium, 1-((2beta,3alpha,5alpha,16beta,17beta)-17-(acetyloxy)-3-hydroxy-2-(4-morpholinyl)androstan-16-yl)-1-(2-propenyl)-, bromide" OR esmeron OR esmerone OR "org 9426" OR org9426 OR zemuron)	#6 AND #11	#6 AND #11
#13	#8 OR #9 OR #10 OR #11 OR #12	#8 OR #9 OR #10 OR #11 OR #12	#8 OR #9 OR #10 OR #11 OR #12		
#14	#7 AND #13	#7 AND #13	#7 AND #13		
#15	(Animals[mh] NOT Humans[mh]) OR Models, Animal[mh:noexp] OR Disease Models, Animal[mh] OR Animal Experimentation[mh]	(animal/exp NOT human/exp) OR 'animal model'/exp OR 'animal experiment'/exp OR 'animal cell'/de OR 'animal tissue'/de OR 'in vitro study'/de OR 'nonhuman'/de			
#16	#14 NOT #15	#14 NOT #15			

Table S2. Summary of this review

ESRD patients’ changes in this review
Delayed recovery to a TOF ratio of 0.9
Decreased clearances of both rocuronium and sugammadex
Increased remaining plasma concentrations of both rocuronium and sugammadex
No changes on the incidence of recurrence of NMB, inadequate recovery of neuromuscular function
Longer retention time of sugammadex-rocuronium complex, but effectively removed through hemodialysis within 24-48 hours after surgery
